# Multi-omics analysis in suspected hereditary breast and ovarian cancer cases reveals novel candidate susceptibility factors

**DOI:** 10.1038/s41523-026-01003-1

**Published:** 2026-06-30

**Authors:** B. Aldrige Allister, Winfried Hofmann, Jonathan L. Lühmann, Björn Sander, Lena Wendeburg, Gunnar Schmidt, Bernd Auber, Susanne Morlot, Hannah Wallaschek, Nataliya di Donato, Brigitte Schlegelberger, Monika M. Golas, Doris Steinemann

**Affiliations:** 1 Department of Human Genetics, Hannover Medical School, Hannover, Germany; 2 Institute of Pathology, Hannover Medical School, Hannover, Germany; 3 Institute of Human Genetics, University Hospital Augsburg and Faculty of Medicine, University of Augsburg, Augsburg, Germany; 4 Comprehensive Cancer Center Augsburg, University Hospital Augsburg, Augsburg, Germany

**Keywords:** Cancer, Computational biology and bioinformatics, Genetics, Molecular biology, Oncology

## Abstract

More than 80% of patients who meet clinical criteria for hereditary breast and ovarian cancer (HBOC) do not show pathogenic variants in *BRCA1, BRCA2*, and other diagnostically consented core genes. We hypothesized that variants in further DNA repair genes, cryptic genomic alterations, or polygenic factors may explain HBOC risk. We studied 134 patients with breast cancer and/or ovarian cancer by the use of whole genome sequencing (WGS), whole transcriptome sequencing (WTS), optical genome mapping (OGM), and mobile element analysis. We identified (likely) pathogenic variants in DNA repair genes in 18 patients, including several RECQ helicase and other DNA repair genes, an intragenic *FANCM* deletion, and six rare mobile element insertions (MEIs) in DNA repair genes. Incorporating PRS306 shifted estimated lifetime breast cancer risk by ≥5 percentage points in a subset of women (*n* = 75), including carriers of rare variants in DNA repair genes, resulting in both upward and downward risk estimation.

## Introduction

Hereditary breast and ovarian cancer (HBOC) is an autosomal-dominant condition accounting for approximately 5-7% of breast cancer (BC) cases and 10-15% of ovarian cancer (OC) cases^1,2^. *BRCA1* and *BRCA2* are the best-characterized genes implicated in HBOC predisposition^3^. Individuals carrying pathogenic or likely pathogenic variants (LPV/PV) in these highly penetrant genes have a substantially increased risk of developing BC and/or OC^4,5^. Currently, the German Consortium for Hereditary Breast and Ovarian Cancer (GC-HBOC) recommends the analysis of 13 core genes (*ATM*, *BARD1*, *BRCA1*, *BRCA2*, *BRIP1*, *CDH1*, *CHEK2*, *PALB2*, *PTEN*, *RAD51C*, *RAD51D*, *STK11* and *TP53)* via next-generation sequencing (NGS)^6^. However, more than 80% of patients test negative for LPV/PV in these genes^7^, suggesting the contribution of other factors.

Given the function of *BRCA1*, *BRCA2*, and most of the other HBOC core genes in DNA repair, additional DNA repair genes could potentially contribute to cancer risk in high-risk HBOC patients who remain uninformative after routine genetic testing. In line with this hypothesis, several studies have implicated LPV/PV in DNA repair genes as being associated with increased risk of HBOC. Girard et al. (2018) presented an association between LPV/PV in *FANCI*, *MASTI*, *POLH*, and *RETL1* genes and BC susceptibility^8^. Similarly, Carvalho et al. (2020) reported two missense variants in *ABRAXAS1* and *PMS2* that were suggested to be associated with HBOC^9^. Felicio et al. (2021) identified a rare missense variant in *FAN1* in two unrelated families and observed loss of heterozygosity (LOH) in *FAN1* in one BC^10^. More recently, Aguilar et al. (2025) reported PVs in *MUTYH*, *CDKN2A*, and *RAD50*, among Mexican patients with hereditary BC^11^. Additionally, a nonsense variant in *BLM* (p.Gln548*), the gene linked to Bloom’s syndrome, was suggested to be associated with BC risk in Slavic populations^12^.

These reports point to the growing genetic heterogeneity of HBOC, indicating the need for increasingly comprehensive diagnostic approaches. Over the past few decades, routine genetic diagnostics have undergone significant advancements, evolving from single-gene testing to NGS-based approaches, including targeted panels, whole exome sequencing (WES), and, more recently, whole genome sequencing (WGS). While WES can readily detect smaller variants, such as single-nucleotide variants (SNVs) and insertions/deletions (indels), structural variants (SVs) are often challenging to identify using short-read-based methods. Optical genome mapping (OGM) is a DNA-labeling-based method developed to detect SVs with high resolution and sensitivity. Together, WGS and OGM provide enhanced detection and characterization of copy number variants (CNVs) and SVs^13,14^.

Noncoding variants such as deep intronic mobile element insertions (MEIs) often remain undetected in routine diagnostics^15^. Mobile elements, or transposable elements, are repetitive genetic sequences scattered throughout eukaryotic genomes, known for their unique ability to move into new genomic locations^16^. These mobile elements can be detected in WES/WGS data using specialized computational tools^17–19^. MEIs have gained attention as potential drivers that contribute in the development of genetic disorders and cancer. We and others detected and validated disease-causing MEIs in the following genes: *ATM*, *APC*, *AVPR2*, *BRCA2*, *CC2D2A*, *COL6A2*, *NIBPL*, *NKX2-1*, *TTN*, and *USH2A* in a rare diseases cohort (Solve-RD) ^20,21^. Torene et al. (2020) reported 14 MEIs classified as LPV/PV in a prospective cohort of clinical exome samples; in 13 cases, the variants were consistent with the patients’ phenotype^17^.

In addition to monogenic causes, polygenic events represent further determinants of HBOC risk. Polygenic risk scores (PRS) are increasingly used to estimate an individual’s risk of developing BC, thereby informing prevention strategies and intervention. There is growing support for incorporating PRS into clinical practice for BC and contralateral BC (cBC) risk evaluation^22–26^. So far, PRS for BC have been predominantly developed and validated in populations of European ancestry^27^. Several versions of PRS for BC have been suggested to date, including PRS_SNP18, PRS_SNP22, PRS_SNP24, PRS_SNP32, PRS_SNP77, PRS_SNP83, PRS_SNP86, PRS_SNP143, PRS_SNP313, and PRS_SNP3820^27^. One of the PRS that best discriminates between cases and controls is PRS_SNP313^27–30^, with BCAC-313 and BRIDGES_306 being the commonly used implementations in the GC-HBOC centers^31^. Both BCAC-313 and BRIDGES_306 are specific versions of PRS_SNP313: BCAC-313 represents the standard 313-SNP model developed by the Breast Cancer Association Consortium (BCAC), while BRIDGES_306 is a modified version of BCAC-313 that excludes several SNPs that could not be reliably analyzed by NGS^31^.

To address the genetic basis of disease in unresolved HBOC patients, this study integrates multiple complementary approaches such as WGS, WTS, and OGM, to identify risk variants. We analyzed a highly selected cohort of 134 HBOC patients, focusing on 238 known DNA repair genes. In addition, PRS analyses were used to assess the cumulative impact of PRS on HBOC risk within the cohort.

## Results

**Fig. 1:**
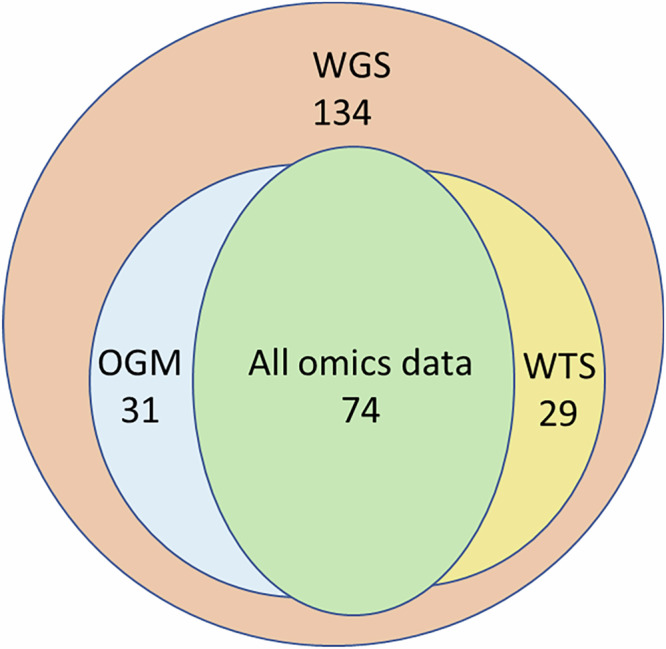
Overview of the multi-omics data for all patients studied herein. Whole genome sequencing (WGS) was performed for all 134 patients. Of these, 103 patients also had whole transcriptome sequencing (WTS) data, and 105 had optical genome mapping (OGM) data. Complete multi-omic data (WGS, WTS, and OGM) were available for 74 patients.

**Fig. 2:**
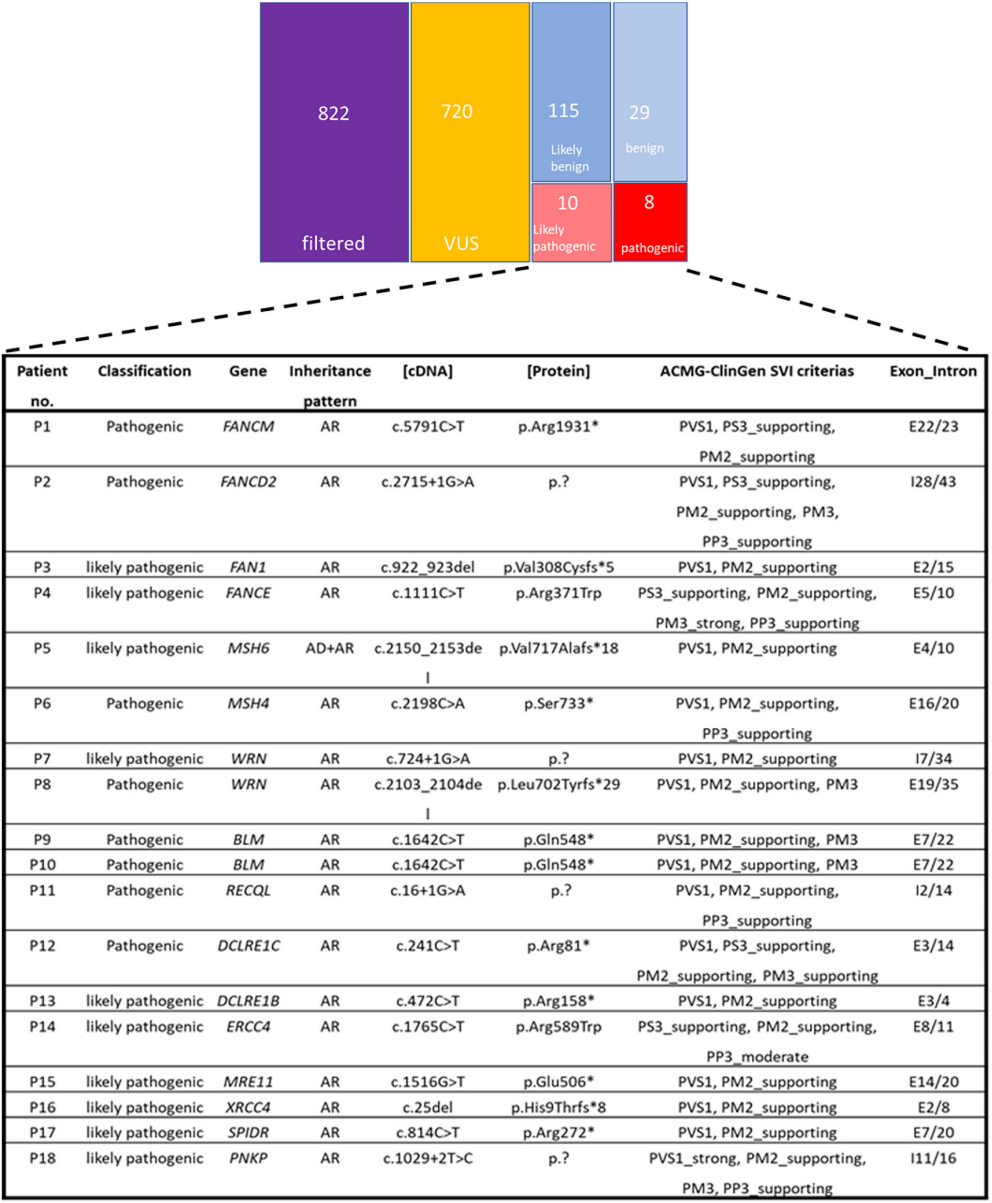
SNVs and indels in DNA repair genes and their classification in our cohort. All variants were classified according to ACMG and ClinGen guidelines^102,108^. Patient numbers correspond to those listed in Table S1. Notation E<#>/<#> or I<#>/<#> indicates the exon/intron number of the variant and the total number of exons (for example, E22/23 denotes a variant in exon 22 from 23 exons in total). AR: autosomal recessive (gene–disease relationship where biallelic LPV/PVs are associated with disease expression), AD: autosomal dominant (gene–disease relationship in which a monoallelic LPV/PV predisposes to disease), E: exon, I: Intron. SVI: Sequence Variant Interpretation, LPV: likely pathogenic variant, PV: pathogenic variant.

Our study cohort includes 134 HBOC high-risk patients, selected based on the criteria outlined above (patient selection). Most of the patients are of Central-Northern European ancestry (125/134), while 9/134 are of different ancestry. The cohort consists of 129 biologically female and 5 biologically male patients, ranging from 27 to 85 years, with 79 individuals under the age of 60 at the time of sampling. WGS was performed for all 134 patients. Additionally, we generated WTS data for 103/134 patients and OGM data for 105/134 patients. A total of 74 patients have data available from all three platforms: OGM, WGS, and WTS (Fig. 1). In concordance with previous diagnostic testing, no PV/LPV was detected in any of the 13 HBOC core genes. Notable genetic results were identified in 24 patients, with their clinical information summarized in Table S1. There are 18 LPV/PVs detected in 18 patients (P1-18) using WGS (Fig. 2, Table S1). A likely pathogenic intragenic SV was detected in one patient (P19), and six rare MEIs were detected in six patients (P1, P20-24). Patient P1 was identified with both a nonsense variant and a rare MEI (Fig. 2, Table 1 and S1).

**Table 1:** List of identified Mobile element insertions (MEIs)

Gene	MEI-Type	Location	Dfam-ID	Identity	Annotation	frequency	Patient no.
*MSH4*	Alu	I17/19	DF000000058.4	AluYc3	g.75,894,801_75,894,802insAlu	1	P20
*MTOR*	Alu	I6/57	DF000000056.4	AluYb9	g.11,253,687_11,253,688insAlu	1	P21
*POLB*	Alu	I2/13	DF000000053.4	AluYa5	g.42,341,720_42,341,721insAlu	1	P22
*POLK*	L1	E15/15	DF000000226.4	5’ end of L1	g.75,598,292_75,598,293insL1	1	P23
*SPIDR*	L1	I8/19	DF000000316.4	ORF2 of L1	g.47,564,681_47,564,682insL1	1	P1
*TP63*	Alu	I3/13	DF000000056.4	AluYb9	g.189,758,057_189,758,058insAlu	1	P24

Table 1 includes the following information: gene, MEI-type, genomic location within the gene: E<#>/<#> or I<#>/<#> indicate the exon/intron number of the variant and the total number of exons (for example, I17/19 denotes a variant in intron 17 from 19 exons in total)., MEI unique identifier according to Dfam database, identity of the soft-clipped sequences in Dfam queries, nomenclature, number of observations within the cohort of 134 patients, and patient number.

### Variants in DNA repair genes

In total, we detected 882 SNVs/indels in DNA repair genes using WGS. Among these, 8 variants were classified as pathogenic, 10 as likely pathogenic, 720 as variants of unknown significance (VUS), 115 as likely benign, and 29 as benign (Fig. 2).

The LPV/PV were identified in genes associated with Fanconi anemia (*FANCD2*, *FANCE*), DNA mismatch repair (*MSH4*, *MSH6*), the RecQ helicases (*WRN*, *BLM*, *RECQL*), DNA crosslink repair (*DCLRE1B*, *DCLRE1C*), and other genes contributing to DNA repair (*FAN1, FANCM, MRE11, SPIDR*, and *PNKP*). The nonsense variants detected include *FANCM* p.Arg1931*, *MSH4* p.Ser733*, *BLM* p.Gln548*, *DCLRE1C* p.Arg81*, *DCLRE1B* p.Arg158*, *MRE11* p.Glu506* and *SPIDR* p.Arg272*. Of these, the *FANCM* truncation variant was identified in patient P1, who was diagnosed with bilateral TNBC. The *BLM* p.Gln548* variant was detected in two unrelated patients, patient P9 and P10, both of whom were diagnosed with Luminal-A BC (ER+, PR+, and HER2-). Frameshift variants were detected in *FAN1* (p.Val308Cysfs*5), *MSH6* (p.Val717Alsfs*18), *WRN* (p.Leu702Tyrfs*29), and *XRCC4* (c.25del). The missense variants identified were *FANCE* p.Arg371Trp and *ERCC4* p.Arg589Trp (Fig. 2).

**Fig. 3:**
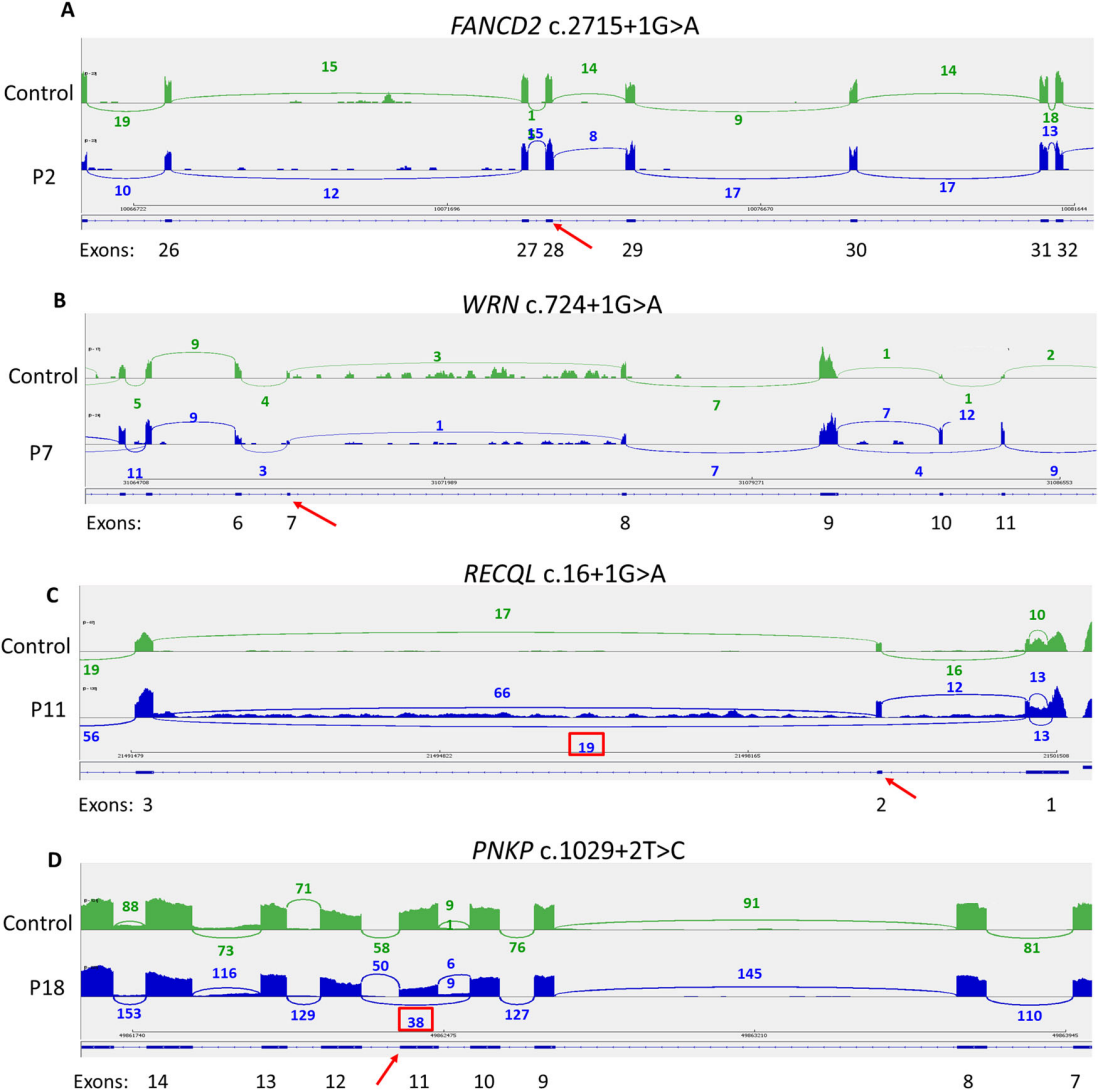
WTS-based analysis of four splice variants in DNA repair genes. Sashimi plots generated in IGV illustrate splicing events: **A** *FANCD2* c.2715+1 G > A at intron 28 of 43, detected in patient P2. **B** *WRN* c.724+1 G > A at intron 7 of 34, detected in patient P7. **C** *RECQL* c.16+1 G > A at intron 2 of 14, detected in patient P11, resulting in exon 2 skipping. **D** *PNKP* c.1029+2 T > C at intron 11 resulting in exon 11 skipping in P18. Control (green plot), patient (blue plot). Exon-spanning reads are shown by connected lines, and the number above gives the number of reads for the connections. Red boxes mark the reads for the exon skipping event. The location of the splice variants is marked by red arrows.

**Fig. 4:**
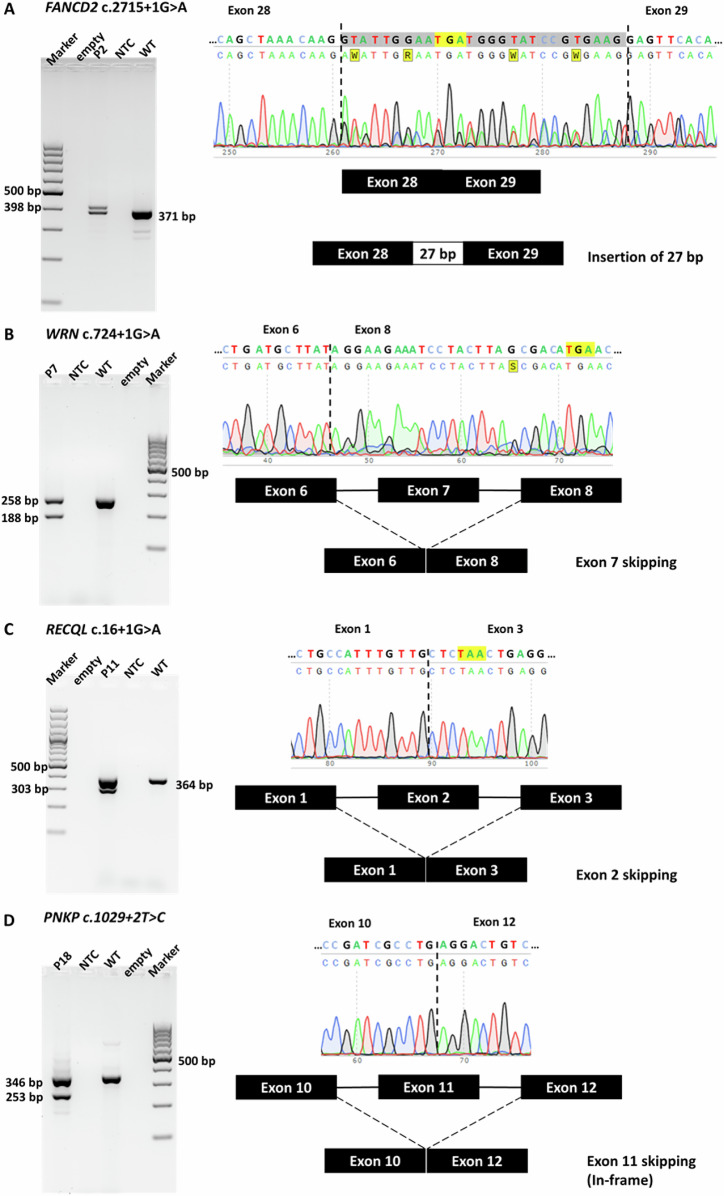
RT-PCR and Sanger-sequencing analysis of four splice variants in DNA repair genes. **A** *FANCD2* c.2715+1 G > A detected in patient P2. **B** *WRN* c.724+1 G > A detected in patient P7, **C** *RECQL* c.16+1 G > A detected in patient P11, **D** *PNKP* c.1029+2 T > C detected in patient P18. Left panel: 2.5% Agarose gel electrophoresis showing RT-PCR products. Marker: DNA ladder 100 bp/100 bp plus; NTC: negative template control; WT: wild type. Right panel (upper part): Sanger sequencing electropherogram of the aberrant PCR product. Interpretations of the Sanger sequencing results are presented above each electropherogram. Dashed lines indicate exon boundaries. **A** inserted sequence is marked grey. **A**–**C** Premature termination codons are marked in yellow. Right side (lower part): schemes how the exons are spliced in the secondary PCR product of the patients. Black box: exons, white box: intron retention, line: introns, dash: splicing.

WTS was performed to assess the functional consequence at the transcript level, specifically in the likely pathogenic/pathogenic splice variants identified in *FANCD2*, *WRN*, *RECQL* and *PNKP*. *FANCD2* c.2715+1 G > A (p.?) was detected in patient P2 with Luminal-B-BC (ER+, PR- and HER2-) contralateral to an unknown type of BC. While the predicted exon 28 skipping event was not detected in WTS from patient 2 (Fig. 3A), RT-PCR confirmed that the *FANCD2* c.2715+1 G > A variant leads to an alternative splicing event. As shown in Fig. 4A, in addition to the 371 WT fragment, a longer fragment of 398 bp was observed. Sanger sequencing of this product showed the retention of 27 bp from intron 28 (c. 2715 + 1_c2715 + 27). This intron retention introduced a premature termination codon at the 4th codon downstream of the exon 28 3’-splice site (Fig. 4A).

The second splice variant, *WRN* c.724+1 G > A (p.?) was identified in patient P7, who presented with bilateral BC (bBC), diagnosed as one Luminal-A and one HER2-positive tumor. Also, in this patient, WTS data did not show the expected isoform in the Sashimi plot, i.e., exon 7 skipping (Fig. 3B). However, RT-PCR analysis demonstrated that the c.724+1 G > A variant results in an aberrant 188 bp product. Sanger sequencing of this product confirmed the exon 7 skipping event, resulting in a premature termination codon (Fig. 4B).

A third splice variant, *RECQL* c.16+1 G > A (p.?), was identified in patient P11 diagnosed with Luminal-A-BC contralateral to an unknown type of BC. The WTS analysis indicated that this variant induces skipping of exon 2 in *RECQL* (Fig. 3C). RT-PCR showed an aberrant transcript of 303 bp. By Sanger sequencing we confirmed that the alternative transcript lacked exon 2, resulting in a premature termination codon (Fig. 4C).

Finally, the splice site variant *PNKP* c.1029+2 T > C (p.?) was detected in patient P18 with BC of unknown subtype. WTS analysis indicated that this variant causes skipping of exon 11 in *PNKP* (Fig. 3D). This splicing alteration was confirmed by RT-PCR, which showed at the cDNA level a 253 bp product. This in-frame exon 11 skipping event was confirmed by Sanger sequencing (Fig. 4D). The exon skipping disrupts the PNKP phosphatase and kinase domains.

### Enrichment analysis

All identified variants are rare or ultrarare (Table S2). Several LPV/PVs, including the *FANCE*, *WRN*, and *SPIDR* variants, were observed in fewer than 20 individuals among approximately 300,000 controls. These frequencies are at similar carrier frequency levels as the *MSH6* variant we identified here, which is associated with autosomal-dominant Lynch syndrome and serves as a reference point herein. As an exploratory means of evaluating whether observed frequencies in cases could be due to random sampling rather than association with disease, carrier frequencies for each of the fourteen identified variants among non-Finnish European women of our cohort were compared against European non-Finnish controls obtained from gnomAD. For this low-frequency context – where most variants were seen in only one affected individual – variant-level comparisons incorporated a beta-binomial model to approximate statistical uncertainty. This approach provided an estimate of enrichment, with results suggesting increased case frequencies for *FANCE*, *WRN*, *BLM*, *MRE11*, and *SPIDR* relative to controls, in addition to the *MSH6* variant. To further examine potential enrichment, we performed gene-level Firth penalized logistic regression after collapsing ultrarare loss-of-function (LOF) variants. Point estimates from the Firth regression suggested higher frequencies of *FANCE*, *WRN*, and *BLM* variants in cases compared to controls (*P*_adjust_ < 0.05; Table S2).

### Intragenic deletion of 32 kb in *FANCM*

**Fig. 5:**
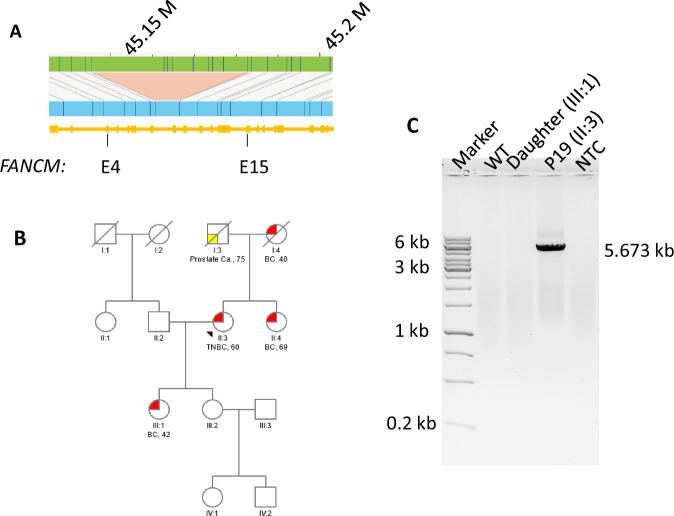
Identification of a 32 kb intragenic deletion in *FANCM* via OMG and WGS. **A** OGM showing a deletion within *FANCM* (marked in red) at chromosomal position 14q21.2 Reference map (green), patient map (blue), vertical black lines: DLE-labels, with concordant labels connected by lines. **B** Pedigree of patient P19. Individual II:3 is P19 marked by a black arrowhead. BC: breast cancer, TNBC: triple negative breast cancer, prostate Ca.: prostate cancer. **C** 1% Agarose gel electrophoresis showing RT-PCR products in P19 (II:3) and her daughter (III:1). A PCR product of about 6000 bp is expected for the deleted allele using forward and reverse primers located at intron 3 and intron 16 of *FANCM*, respectively. Marker: 1 kb DNA ladder, WT (wild type), NTC (negative template control).

OGM was performed on 105 patients from the cohort. A single structural variant was identified: a heterozygous intragenic deletion in *FANCM* spanning exon 4 to 14: (ogm[GRCh38] 14q21.2(45,145,384_45,183,139)x1 (Fig. 5A). The deletion was identified in patient P19, diagnosed with TNBC (Table S1). To validate this result, we conducted WGS and confirmed the 32 kb deletion in the *FANCM* gene (seq[GRCh38] 14q21.2(45,148,718_45,180,718)x1). In patient’s P19 family, there are three other individuals affected by BC, her mother (I:4), her sister (II:4) and one of her daughters (III:1) (Fig. 5B). Upon acquiring blood sample of the daughter III:1 (no information regarding the type of BC available), we performed a segregation analysis via RT-PCR (Fig. 5C). Segregation analysis showed the absence of the *FANCM* deletion in the daughter (III:1).

### Identification of six rare MEIs

*Mobster* analysis was performed on WGS data sets of 134 HBOC patients. Six rare MEIs in DNA repair genes were detected (Table 1).

An Alu element insertion in intron 3 of the *MSH4* gene seq[GRCh38] der(1)ins(1;?)(p31.1;?) NC_000001.11:g.75,894,801_75,894,802insAlu was detected in patient P20, who was diagnosed with TNBC. Analysis of the soft-clipped bases at the left side of the insertion point confirmed the presence of an AluYc3 element (DF000000058.4) (Fig. S2A). In patient P21, who has been diagnosed with Luminal-A BC, an *Alu* insertion in intron 6 of the *MTOR* gene seq[GRCh38] der(1)ins(1;?)(p36.22;?) NC_000001.11:g.11,253,687_11,253,688insAlu was detected. The right side of the insertion point matched AluYb9 (DF000000056.4) (Fig. S2B). Patient P22, also diagnosed with Luminal-A BC, harbored an *Alu* element insertion in intron 2 of the *POLB* gene seq[GRCh38] der(8)ins(8;?)(p11.21;?) NC_000008.11:g.42,341,720_42,341,721insAlu. The right side of the insertion point corresponded to AluYa5 (DF000000053.4) (Fig. S2C).

The L1 element insertion in exon 15 of the *POLK* gene seq[GRCh38] der(5)ins(5;?)(q13.3;?) NC_000005.10:g.75,598,292_75,598,293insL1 was identified in patient P23, who has been diagnosed with OC and Luminal-A BC. The left side of the insertion point was identified as the 5’ end of L1 retrotransposon (DF000000226.4) (Fig. S2D). Patient P1, who was diagnosed with TNBC, carries an L1 element insertion in intron 8 of the *SPIDR* gene, seq[GRCh38] der(8)ins(8;?)(q11.21;?) NC_000008.11:g.47,564,681_47,564,682insL1[1,293]. The right side of the insertion point matched ORF2 of L1 retrotransposon (DF000000316.4) (Fig. S2E). OGM analysis of this patient confirmed this insertion (data not shown). An *Alu* element insertion in intron 3 of the *TP63* gene, seq[GRCh38] der(3)ins(3;?)(q28;?) NC_000003.12:g.189,758,057_189,758,058insAlu was found in patient P24, who has been diagnosed with Luminal-A BC. The left side of the insertion point is AluYb9 (DF000000056.4) (Fig. S2F). There were either no OGM data for the other five MEIs or the size of the insertion was below the detection limit of OGM platform.

### PRS for risk stratification in HBOC patients

**Fig. 6:**
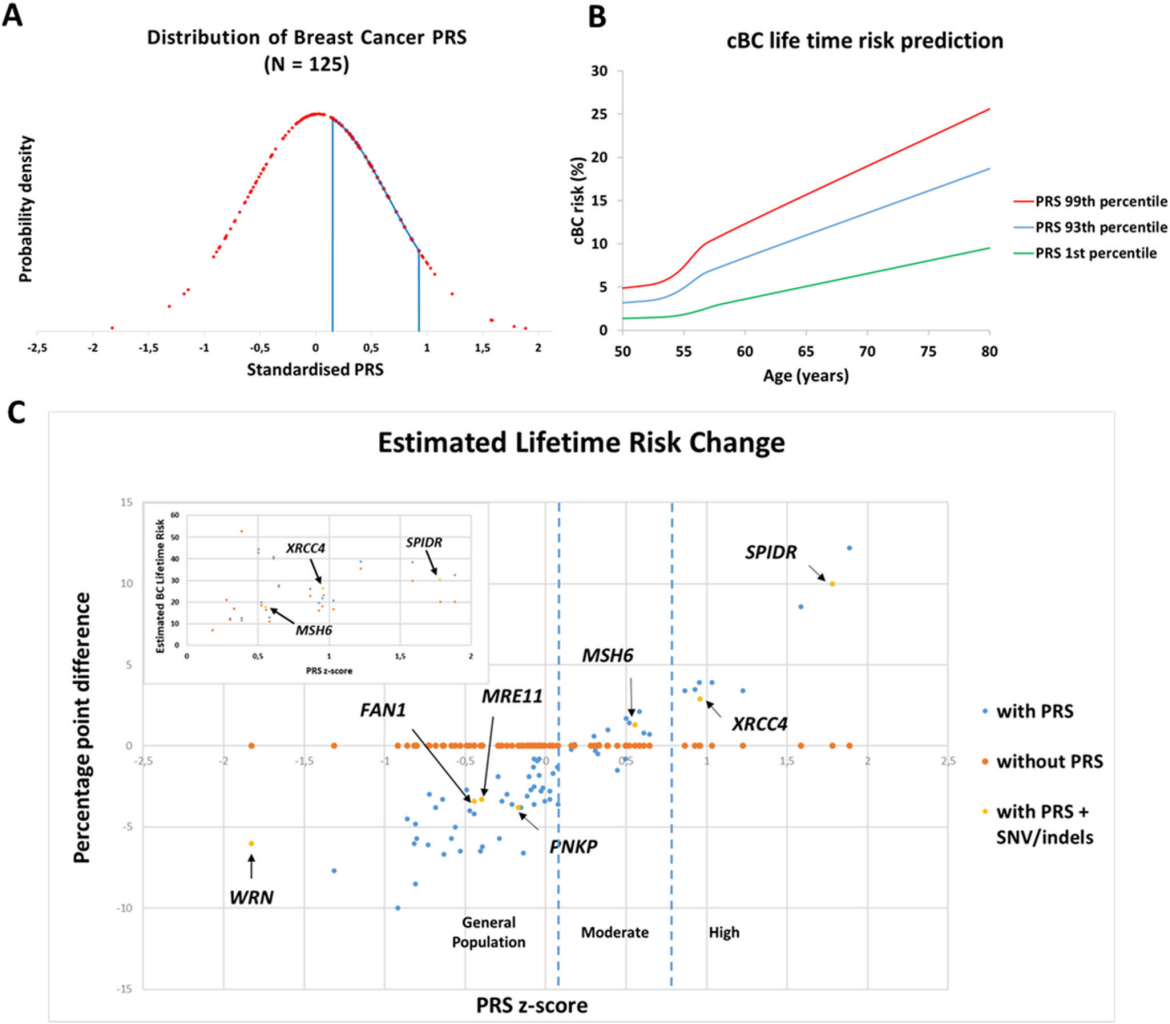
PRS and lifetime cBC/BC risk prediction. **A** Density plot showing the distribution of BC PRS among 125 HBOC patients of Central-Northern European origin (red). The distribution is divided into three PRS categories (class 1, 2, and 3) separated by threshold lines (blue). **B** cBC risk estimation for representative patients from each PRS category: PRS class 3 (99th percentile; red), PRS class 2 (93rd percentile; blue), PRS Class 1 (1st percentile; green). The recorded ages were 47, 47, 48 at first diagnosis for the PRS class 3, class 2, class 1, respectively. Risk was calculated using the CanRisk-tool v3.0.0. BC: breast cancer. **C** Life time BC risk estimation in 75 women. Blue dashed lines indicate the three risk categories according to **A**. Each dot shows the risk difference (percentage points) in estimated lifetime BC risk with (blue) versus without (orange) incorporating PRS_306_, plotted against the PRS_306_ z score. LPV/PV carriers are indicated as yellow dots and with arrows. Inset: Estimated lifetime BC risk for women with a ≥ 0 percentage point difference.

Finally, we calculated the PRS for all 125 HBOC patients of Central-Northern European ancestry using PRS BRIDGES_306. Following classification, 77/125 patients were assigned to the PRS group class 1 (PRS score: −1.828–0.146), 33/125 patients to the PRS group class 2 (PRS score: 0.152– 0.798), and 15/125 patients to the PRS group class 3 (PRS score: 0.859–1.886) (Fig. 6A). In line with the supported patient profiles of the CanRisk tool, analysis of lifetime cBC risk included 75 female patients of Central-Northern European origin. Figure 6B presents the differences in the estimated lifetime risk of developing cBC for representative patients from each PRS group of similar age within our cohort. In a woman diagnosed with breast cancer at age 47, the estimated lifetime risk of developing a contralateral breast cancer by age 80 in the general population is about 16% according to CanRisk. In comparison, a representative patient in PRS class 1 with an age of 48 at first diagnosis (1st percentile) had an estimated lifetime cBC risk of 9.5%, while a representative patient in PRS class 2 with an age of 47 at first diagnosis (93rd percentile) had an 18.7% life time risk. A representative patient in PRS class 3 (99th percentile) with an age of 47 at first diagnosis had a lifetime risk of 25.6%.

Finally, we evaluated the impact of PRS_306_ on the lifetime risk estimation of BC in these 75 patients. Overall, 22 patients (29%) had an estimated lifetime BC risk of ≥ 25%, when PRS_306_ was incorporated (range 4.9–52.8%). In 21 women, inclusion of PRS_306_ resulted in an estimated lifetime BC risk difference of ≥ 0 percentage points (Fig. 6C, inset). Applying a cut-off of 5 percentage point change in risk, 3 of 75 patients showed an increase of risk (8.6–12.2 percentage points) upon adding PRS_306_, whereas 15 patients were reclassified to a lower estimated risk of BC (reduction of 5.0–10.0 percent percentage points; Fig. 6C). These groups included patient P8 carrying the *WRN* variant c.2103_2104 as well as the patients P17 (*SPIDR*, p.Arg272*), P16 (*XRCC4*, p.His9Thrfs*8), P5 (*MSH6*, p.Val308Cysfs*5), and P18 (*PNKP*, c.1029+2 T > C). Taken together, these observations indicate that incorporating PRS_306_ into clinical risk models leads to clinically meaningful upward and downward shifts in estimated lifetime BC risk in a subset of women, including carriers of rare LPV/PV.

## Discussion

A central result of this study is the identification of monoallelic LPV/PVs in genes associated with autosomal-recessive inheritance (*FANCM*, *FANCD2*, *FAN1*, *FANCE*, *MSH6*, *MSH4*, *WRN*, *BLM*, *RECQL*, *DCLRE1C*, *DCLRE1B*, *ERCC4*, *MRE11*, *XRCC4*, *SPIDR*, and *PNKP*) among HBOC patients. Of these, LPV/PVs in *MSH6* are associated with increased cancer risk in the context of Lynch syndrome (monoallelic) and Constitutional Mismatch Repair Deficiency (biallelic)^32^. Other genes, including *FANCD2*, *FANCE*, *WRN*, and *ERCC4* have established roles in the maintenance of genomic stability, DNA repair, and, in case of functional impairment, predisposition to cancer^33–35^.

Emerging evidence suggests that even a monoallelic variant in DNA repair genes, including *FANCM*, *WRN*, *BLM*, *MRE11* and *RECQL* could potentially have biological consequences relevant to cancer predisposition^36–40^. Many of these genes represent RecQ helicase paralogues with well-established roles in autosomal-recessive disorders, and a growing body of experimental and epidemiological data suggests that haploinsufficiency may contribute to tumor susceptibility in certain contexts^40–42^. Along these lines, heterozygous LoF variants in DNA repair genes could compromise the efficiency of critical DNA repair pathways and lower the cellular capacity for tolerating DNA damage, particularly in tissues with high proliferative rates or those exposed to DNA-damaging agents. For example, lymphoblastoid cell lines derived from carriers of heterozygous LoF variants in *WRN* have intermediate sensitivity to genotoxic agents compared to wild-type cells and cells with biallelic *WRN* variants^41^. Biochemically, this intermediate phenotype is related to reduced WRN functional protein levels: Quantitative analyses revealed that *WRN*-heterozygotes retain approximately 50% of wild-type WRN protein levels and its helicase activity^43^. Residual helicase activity in heterozygous carriers may thus provide partial compensation initially, but over time may be associated with increased accumulation of DNA damage, potentially resulting in the development of neoplastic disease.

*RECQL* has been identified to contribute to DNA double-strand break (DSB) repair and the maintenance of genomic stability^44^. Previous work showed that RECQL-deficient cells or knockout mice displayed chromosomal instability and increased DNA damage^45^. Moderate siRNA-based knockdown of *RECQL* in BC cells results in a significant increase in DNA double-strand breaks^40^. Of note, BC cells from *RECQL* PV carriers did not show loss of heterozygosity (LOH), suggesting that *RECQL*-associated tumorigenesis may result from haploinsufficiency, where a single functional copy of *RECQL* is insufficient to maintain DNA repair upon DSB accumulation^45^.

We also identified LPV/PV in *BLM*, the third RecQ helicase gene. Monoallelic loss-of-function variants in murine models lead to earlier and more frequent tumor development, suggesting that partial loss of *BLM* function may compromise genomic stability^42^. Given BLM’s critical role in dissolving Holliday junctions and other complex DNA structures^46^, haploinsufficiency could reduce the cellular capacity to suppress mutagenic recombination and to appropriately repair stalled replication forks^47^. Moreover, heterozygous carriers of the *BLM* variant identified in two patients with hormone receptor–positive, HER2-negative BC in our cohort have been reported to have an elevated BC risk in some populations, particularly among individuals of Slavic descent^12^, but not an increased risk for OC^48^.

Biallelic LoF variants in Fanconi anemia (FA) genes – components of the DNA damage response^49,50^ – are associated with bone marrow failure, short stature, and predisposition to cancer^51,52^. Individuals with heterozygous LPV/PVs in FA-genes – next to *BRCA1* (*FANCS*) and *BRCA2* (*FANCD1*) – may face increased cancer risk, including BC^53,54^. Heterozygous *FANCM* variants have been linked to cancer susceptibility, including BC, with a tendency to develop the ER- and TNBC subtype of BC^55,56^. The *FANCM* variant c.5791 C > T (p.Arg1931*) detected in patient P1 diagnosed with TBNC has been previously described by Peterlongo et al. (2015) as a risk factor for familial BC^57^. This variant has been associated with TNBC in subsequent studies^36,55,58^. However, a recent study by Tervasmäki et al. (2025) suggests that *FANCM* c.5791 C > T does not associate with the overall BC risk in the northern Finnish population, but shows a non-significant association with TNBC^59^. In patient P19, diagnosed with TNBC, a likely pathogenic intragenic deletion in *FANCM*, spanning exons 4 to 14, was observed. This deletion was not inherited by patient P19’s daughter, who also developed BC, although data on the subtype were unavailable.

Next, the *FANCD2* c.2715+1 G > A was detected in patient P2 with Luminal-B-BC (ER+, PR-, and HER2-), contralateral to an unknown type of BC. This variant has been previously reported by Mantere et al. (2017) in an early-onset TNBC patient and was proposed as a potential BC risk factor within the northern Finnish patient cohort^60^. The splice variant has also been reported in patients with FA^61,62^. The *FANCE* c.1111 C > T (p.Arg371Trp) variant detected in patient P4 diagnosed with OC has been reported in a patient with bone marrow failure syndrome^63^ and in Brazilian patients with pancreatic adenocarcinoma^64^. The *FANCE* Arg371Trp lies in a position that has been shown to be essential for interaction with FANCD2^65^. The missense variant Arg371Trp may destabilize the protein structure of FANCE and thereby inhibit its interaction with FANCD2, required for FA core complex assembly^66–68^.

Data on an association between BC and some other DNA repair genes, such as *SPIDR*, *PNKP* and *MRE11* are limited. SPIDR was previously identified to interact with RAD51 and BLM, both involved in maintaining genomic stability^69^. A homozygous c.814 C > T (p.Arg272*) variant in *SPIDR* was recently reported in an Indian patient with primary ovarian insufficiency. Patient lymphocytes showed an increase of mitomycin C-induced chromosomal instability with aberrant metaphases compared to controls, indicating impairment of the RAD51 pathway^70^. Here, we identified this variant in patient P17 with luminal A-BC.

Patient P18 was diagnosed with BC (unknown subtype) and carries the heterozygous *PNKP* c.1029+2 T > C variant. This splice variant was previously reported in a patient with developmental delay and microcephaly (OMIM #613402), who was compound heterozygous for this variant and *PNKP* c.151 G > C (p. Val51Leu), each variant inherited from one parent. The mother of the patient, who also carries the splice variant, has no documented history of cancer^71^. Rudenskaya et al. (2019) also reported this splice variant in a compound heterozygous state in a patient with Ataxia with oculomotor apraxia type 4 (AOA4, OMIM # 616267)^72^. We showed that the splice variant *PNKP* c.1029+2 T > C causes an in-frame deletion of exon 11 (p.Phe313_Pro343del), which shortens the Phosphatase domain of PNKP (191 amino acids) by 31 amino acid residues and may lead to reduced DNA repair capacity, causing genomic instability^73,74^.

Biallelic pathogenic germline variants in *MRE11A* lead to autosomal recessive ataxia-telangiectasia-like disorder (OMIM #604391), a condition that follows a milder and slower course compared to classical ataxia-telangiectasia (OMIM #208900)^75^. Truncating variants in *MRE11A*, including *MRE11* c.1516 G > T (p.Glu506*) have been occasionally reported in ClinVar. However, supporting evidence for their pathogenicity, such as segregation or functional analysis, is often missing^76–78^. Amongst others, the truncating *MRE11* variant was detected in two unrelated French-Canadian HBOC patients as well as in a case-control study involving approximately 2500 patients from the French-Canadian population of Quebec. In line with its role as activator of ATM^79^, a member of the DNA damage response pathway whose LOF typically does not promote a homologous recombination deficiency (HRD) phenotype^80^, mutational signature analysis revealed that the BC did not exhibit HRD signature^76^.

The presence of variants in genes such as *FANCE*, *WRN*, and *SPIDR* at very low carrier frequencies in controls, comparable to that of a well-established pathogenic *MSH6* variant, supports enrichment among affected individuals. However, the small number of variant observations limits statistical power. To address the uncertainty inherent in this low-frequency context, we applied a beta-binomial model, which indicated enrichment of several variants (notably in *FANCE, WRN, BLM, MRE11*, and *SPIDR*). At the gene level, Firth regression analysis suggested higher frequencies of *FANCE, WRN*, and *BLM* variants in cases compared to controls, with these three genes reaching statistical significance in both analyses. Yet, these results are exploratory. Overall, these results provide support for the involvement of rare variants in these genes in disease susceptibility, as also reflected by functional data, but larger cohorts will be required for confirmation.

Overall, heterozygous LPV/PVs in non-HBOC core genes involved in DNA repair may increase the risk of developing cancer, though the risk may not be as high as from moderate to high-risk monogenic cancer susceptibility genes such as *BRCA1*, *BRCA2*, or *ATM*. In general, low penetrance variants and environmental factors, such as lifestyle or exposure to carcinogens, may contribute to the large variability of penetrance observed in moderate to high risk monogenic cancer susceptibility genes^81,82^. Modifier variants may contribute to cancer risk by subtly increasing the probability of accumulating additional genetic alterations and promoting genomic instability over time, and their impact on cancer may be best understood within the broader context of polygenic and environmental factors^38,83,84^.

The extended DNA repair gene set selected in this study should be regarded as a discovery framework enabling systematic exploration of rare variants beyond the established HBOC core genes. In clinical practice, expansion of reportable genes is likely to proceed gradually as evidence accumulates, balancing the potential benefits of broader testing and improved risk stratification against the increased likelihood of VUS and the associated interpretive complexity for patients and clinicians.

Of note, our analysis of WTS data from whole blood showed low expression of several HBOC core genes, including *BRCA1*, *BRCA2*, *PALB2* as well as other DNA repair genes such as *FANCD2* and *WRN*, which interfered with or precluded effective analysis at the RNA level. In contrast, the expression of genes such as the HBOC core genes *ATM*, *PTEN*, and *TP53*, and some further DNA repair genes such as *PNKP* and *RECQL*, was sufficiently high to be analyzed by WTS. This suggests that WTS analysis of whole blood of HBOC patients may not be a suitable material to analyze the expression profile of these genes. Alternative tissues, particularly breast and ovarian tissue, would represent more suitable sources for RNA extraction.

Beyond SNVs and indels, we tested for the potential contribution of MEIs to HBOC predisposition. We identified six MEI insertions in DNA repair genes: Alu element insertions in *MSH4* and *POLB*, genes involved in DNA repair and recombination^85,86^, *MTOR*, contributing to the regulation of cell growth, metabolism, and signaling^87,88^, and in *TP63*, a p53 family member involved in DNA damage response by promoting cell cycle arrest^87,89^. L1 element insertions we detected in *POLK* and *SPIDR*. *POLK* is known to be involved in DNA repair at stalled replication forks following DNA damage^90,91^, and *SPIDR* to regulate homologous recombination by interacting with *RAD51* and to promote repair of DNA double-strand breaks^69,92^. Upon characterizing the six detected MEIs on transcript level, WTS and RT-PCR along the neighboring exons around the insertion site, we did not observe alternative splicing events caused by the insertion of these elements (data not shown). To our knowledge, these MEIs have not been reported before.

The implementation of PRS into BC diagnostics has recently been proposed to improve risk prediction and refine individualized surveillance strategies ^21–25^. European guidelines recommend that women become eligible for mammography screening at the age of 50, corresponding to the age at which the incidence of BC becomes clinically significant in the general population^93^. While the clinical implementation of PRS is still evolving, numerous studies support the use of PRS for BC risk estimation in personalized medicine^27–31,94^. At present, there is no universally accepted standard for defining and categorizing individuals into high (class 3), moderate/average (class 2), and low/general population (class 1) PRS groups. In our study, we stratified patients into PRS categories according to the PRS percentile thresholds proposed by Padrik et al.^22^.

Mavaddat et al. (2018) observed that women in the highest PRS strata reach risk levels comparable to the general population several years earlier (calculation performed using PRS313 and data from 79 studies conducted by BCAC)^28^. Beyond this shift in age‑specific risk, we observed that incorporating PRS_306_ into risk estimation led to clinically relevant modification of lifetime BC risk in a subset of women: 18/75 patients experienced risk changes of ≥ 5 percentage points, including both upward and downward shifts. This modification affected not only women without identified LPV/PVs in the expanded gene panel tested herein but also carriers of rare variants in DNA repair genes such as *WRN, SPIDR, XRCC4, MSH6* and *PNKP*.

These observations suggest that PRS may help to refine risk estimates derived from established models and, in the future, could contribute as one of several factors in clinical decision-making, for example, when considering intensified surveillance, chemoprevention, or risk-reducing surgery in selected patients. At the same time, our cohort is relatively small and predominantly consists of women of Central‑Northern European ancestry, and the clinical thresholds for acting on PRS‑modified risk estimates are not yet standardized. Larger, ancestrally diverse validation studies with prospective follow‑up are needed to define robust cut‑offs for PRS‑informed reclassification, clarify the contribution of individual “non‑canonical” genes such as *WRN* to risk models, and evaluate whether PRS‑guided management ultimately improves patient‑relevant outcomes. Collectively, our data support the concept that heterozygous LPV/PVs in autosomal-recessive DNA repair genes may compromise function along a continuum, where reduced activity may create a permissive landscape for genomic stress, leading to an accumulation of DNA damage. Such a model has broader relevance for understanding cancer predisposition in carriers of heterozygous variants in these genes. Further research, including functional studies and large-scale population studies, will be essential to clarify the precise contribution of these heterozygous variants to cancer risk and to inform clinical management strategies for carriers. In this context, comprehensive genomic approaches such as WGS, with additional assays introduced in a tiered diagnostic strategy for selected unresolved patients, may provide a framework to detect and characterize such variants in clinical practice.

## Methods

### Patients

All individuals included gave their informed written consent to participate in our study. The study has been performed in accordance with the Declaration of Helsinki and was approved by the local ethics committee (Hannover Medical School, Ethic votes: Nr. 4121 and extension Nr. 8657_BO_K_2019). The clinical data collected during genetic counseling, including birth cohort and family history, were integrated into the Breast and Ovarian Analysis of Disease Incidence and Carrier Estimation Algorithm (BOADICEA) model 7.3.2, version 0.6.0, via the CanRisk-tool v3.0.0. (https://www.canrisk.org/canrisk_tool/)^95–97^. The estimated risk of a patient carrying an inherited LPV/PV in any of the genes *ATM*, *BARD1*, *BRCA1*, *BRCA2*, *BRIP1*, *CHEK2*, *PALB2*, *RAD51C*, or *RAD51D* is calculated through the CanRisk tool as an LPV/PV carrier probability (PVCP)^95–97^. The selection criteria of our research cohort are the following:

1. The index patient has been diagnosed with BC at the age of 50 or younger, and/or triple negative BC (TNBC) at the age of 65 or younger, and/or OC below 80.
2. The index patient has at least one family member affected by BC and/or OC.
3. The index patient has tested negative for LPV/PV in all 13 HBOC core genes, as determined by diagnostic procedures (panel or exome sequencing).
4. The index patient has been calculated for pathogenic variant carrier probability (PVCP) ≥ 10%.
5. In exceptional cases, we also included patients under 80 years whose history indicated a pattern of cancer suggestive of hereditary cancer predisposition. For example, patients with a PVCP below 10% were included if their family history revealed multiple cases of breast, ovarian, pancreas and/or prostate cancer among 1st and 2nd degree relatives.

Criteria 1-4 are the general requirements that must be fulfilled for the patient selection. Criterion 5 is considered when at least criteria 2 and 3 are fulfilled.

### Controls

Data on non-Finnish European female individuals were extracted from gnomAD v4.1.0 and used as controls^98^. Variant extraction was performed using Hail (https://github.com/hail-is/hail). Only high-confidence variants were considered. Applied filters included: female sex, non-Finnish European ancestry, PASS, MAF < 0.001, absence of homozygous carriers, and VEP impact classified as HIGH or, for MODERATE impact, variants reported as likely pathogenic or pathogenic in ClinVar. Variants with conflicting pathogenicity, unknown significance, or classified as a risk factor in ClinVar were excluded.

### DNA and RNA isolation

DNA was isolated from frozen EDTA blood using QIAamp DNA Blood Mini Kit (Ref. 51104) according to protocols provided by the manufacturer (Qiagen, Hilden, DE). RNA was isolated from frozen EDTA blood using Nucleospin RNA blood Kit (Ref. 740200.50) according to the manufacturer’s instructions (MACHEREY-NAGEL, Düren, DE). DNA and RNA samples were submitted to the Competence Centre for Genomic Analysis (CCGA), Kiel, Germany, where the sequencing was performed according to standardized protocols (https://ccga.uni-kiel.de).

### NGS

Sequencing was performed using Illumina-based methods, with 30× coverage for WGS and 25 million reads/sample for WTS. Reads were aligned to the human reference genome GRCh38/hg38. In total, we performed WGS and WTS on 134 and 103 patients, respectively.

### Validation by RT-PCR and Sanger sequencing

Following RNA extraction, cDNA synthesis was carried out using the High-Capacity cDNA Reverse Transcription kit (Applied Biosystems, Waltham, MA, US). Primers were designed using the Primer-BLAST tool (https://www.ncbi.nlm.nih.gov/tools/primer-blast/)^99^ and ordered from Metabion (Planegg, DE). Reverse transcription-polymerase chain reaction (RT-PCR) was performed using GoTag® G2 DNA Polymerase kit according to protocols provided by the manufacturer (Promega, Madison, WI, US). DNA from the PCR product was extracted using NucleoSpin Gel and PCR Clean-up kit (MACHEREY-NAGEL, Düren, DE) and sent to Microsynth (Göttingen, Germany) for Sanger sequencing.

### WGS data processing

The genomic raw data (fastq-files) were processed and analyzed using the “analyze”- script of our in-house NGS analysis pipeline, Medical Genetics Sequence Analysis Pipelines (megSAP), version 2023_11-315-gc8f66b47 (https://github.com/imgag/megSAP). This pipeline integrates state-of-the-art bioinformatic tools to perform alignment against the GRCh38 human reference genome, variant calling, and annotation. Variant filtering was carried out using the software GSvar (https://github.com/imgag/ngs-bits) (Figure S1) with the following settings: allele frequency ≤ 0.10%, variant quality qual >=200, depth > =0, mapq > =40, strand_bias > =20, filter passed (remove low_conf_region, off-target, low_mappability, mosaic), predicted pathogenic filter min > =1, cuttoff_phylop > =1.6, CADD > = 20, REVEL > = 0.9, alphamissense > =0.56. The detected variants were next filtered based on a curated set of 238 known DNA repair genes from the human genome, including the KAUFFMANN_DNA_REPAIR_GENES.v2024.1.Hs (https://www.gsea-msigdb.org/gsea/msigdb/human/geneset/KAUFFMANN_DNA_REPAIR_GENES.html)^100,101^ and five additional DNA repair genes not listed in this gene set: *PARG*, *FAN1*, *GEN1*, *WDR48*, and *SPIDR*. Hereafter, we refer to this gene set as DNArepair_238 The Variants were then classified according to American College of Medical Genetics (ACMG) and ClinGen sequence variant interpretation guidelines^102,103^.

### MEIs detection

MEI detection was performed by submitting the WGS data of 134 patients to Mobster for analysis (Fig. S1). The detected MEIs were then filtered based on the DNArepair_238 gene set. The soft-clipped base sequences, visualized using Integrative Genomic Viewer (IGV) were submitted to the Dfam 3.7 database (https://dfam.org/home) to identify the subfamily of the inserted element^104^.

### WTS data processing

WTS raw data alignment against the GRCh38 reference genome was performed using the analyze_rna -script of the megSAP pipeline (version 2022_12-22-g8d1a6b6f, https://github.com/imgag/megSAP). Sashimi plots generated from the BAM file in IGV were used to visualize the alternative splicing events as a functional consequence of the splice variants detected by WGS.

### OGM

OGM was performed for 105 patients according to the protocols provided by the manufacturer (Bionano Genomics Inc., San Diego, CA, US). Ultra-high molecular weight DNA (UHMW-DNA) was isolated from frozen EDTA blood of the patients using the Bionano Prep SP Frozen Human Blood DNA Isolation Protocol v2 (Document 30395 rev. B, Bionano Genomics Inc.). The isolated DNA was fluorescently labeled at a specific 6 bp genomic pattern (CTTAAG) using the Direct Label Enzyme (DLE-1), following the Bionano Prep Direct Label and Stain Protocol (Document 30206 rev. G, Bionano Genomics Inc.). Subsequently, the labeled UHMW-DNA was visualized and captured using the Saphyr Genome Imaging Instrument (Bionano Genomics Inc.), resulting in the collection of >1,500 Gbp of data per sample. The molecules detected were filtered to include only molecules exceeding 300 kbp in length, with the summed length of all molecules up to 500 Gbp in total. The data were subjected to de novo assembly and annotation (Solve version 3.7, Bionano Genomics Inc.), and the resulting genome maps were visualized using the Bionano Access Server (Access version 1.7.x, Bionano Genomics Inc.). The recommended filter settings were applied, and only variants present with a frequency of less than 1% in the Bionano Genomics control database were analyzed^105^.

### Pedigree tree

The pedigree tree of patient P19 was made by PROGENY online pedigree tool (https://progenygenetics.com/online-pedigree).

### PRS

As our cohort is composed primarily of individuals of Central-Northern European ancestry, we used BRIDGES_306^31,94^. Nine of the 134 patients are not of Central-Northern European ancestry and were therefore excluded. PRS z-scores and percentiles for all 125 patients were derived from the WGS data and used to calculate the BC lifetime risk using CanRisk-tool v.3.0.0^95–97^. The extracted PRS scores from 125 patients were plotted as a probability density to visualize the distribution. Importantly, CanRisk is not designed to calculate lifetime risk for patients who have already suffered from contralateral BC (cBC), ovarian cancer, male patients, or patients at the age of 80 or older. Therefore, we focused on 75 female BC patients of Central-Northern European ancestry for further PRS analysis. These patients were categorized into three PRS risk groups using the BC PRS percentiles according to the UK National Institute for Health and Care Excellence (NICE) guidelines, as well as recommendations given by Padrik et al. (2025)^22,23,106^.

1. Percentiles 1–79: low/general population risk (class 1).
2. Percentiles 80–97: moderate risk (class 2).
3. Percentiles 98–99: high risk (class 3).

To estimate the effect of PRS on lifetime breast cancer risk using CanRisk, we set patients’ age to 20 years and calculated risks with and without PRS as previously described^107^.

### Statistical analyses

Variant-level and gene-based burden analyses were performed to evaluate the enrichment of rare variants in cases compared to controls. For each variant, the numbers of carriers and non-carriers in both cohorts were tabulated, and enrichment was assessed using a Bayesian beta-binomial framework, implemented via the VGAM R package v1.1.13. Gene-level burden testing utilized Firth penalized logistic regression, carried out with the logistf package v1.26.1, to address rare event characteristics and data separation. Correction for multiple hypothesis testing was performed using the Benjamini-Hochberg false discovery rate procedure to adjust *P*-values. An (adjusted) *P*-value ≤ 0.05 was considered significant.

### Ethics approval and consent to participate

Participants of the study are recruited at the local genetic counselling center using standard procedures approved by the local ethics committee (approval ID 8657 BOK 2019) and have given consent to participate in the study.

## Supplementary information

Supplementary Information

## Data Availability

To protect patient confidentiality, individual-level data cannot be shared. Aggregated data can be provided by the corresponding author upon reasonable request, which should include a brief research proposal. Data sharing will be subject to the completion of the necessary agreements.
