# Supplementary material for: Multi-omics analysis in suspected hereditary breast and ovarian cancer cases reveals novel candidate susceptibility factors
Source: NPJ Breast Cancer. 2026 Jun 30;12:87. doi: 10.1038/s41523-026-01003-1 (PMC13319475; doi:10.1038/s41523-026-01003-1)
Supplement: Supplementary file 1 — Supplementary Information [file 41523_2026_1003_MOESM1_ESM.docx]

**Table S1. Clinical information of patients.**

| **Patient no.** | **Diagnosis** | **Sex** | **Localization** | **Age of diagnosis** | **ER, PR, HER2 status** | **CanRisk-PVCP (%)** |
| --- | --- | --- | --- | --- | --- | --- |
| P1 | BC | female | left | 61 | ER-, PR-, HER2- | 76.83 |
|  | BC |  | right | 69 | ER-, PR-, HER2- |  |
| P2 | BC | female | right | 52 | unknown | 28.44 |
|  | BC |  | left | 55 | ER+, PR-, HER2- |  |
| P3 | BC | female | left | 36 | ER-, PR-, HER2- | 12.83 |
| P4 | OC | female | left | 51 | ER+, PR+ | 18.9 |
| P5 | BC | female | left | 46 | ER+, PR+, HER2+ | 9.83 |
| P6 | OC | female | left | 48 | unknown | 36.57 |
|  | BC |  | left | 66 | ER-, PR-, HER2 unknown |  |
| P7 | BC | female | left | 48 | ER+, PR+, HER2- | 21.66 |
|  | BC |  | right | 48 | ER+, PR-, HER2+ |  |
| P8 | BC | female | right | 42 | ER-, PR-, HER2- | 7.45 |
| P9 | BC | female | left | 79 | ER+, PR+, HER2- | 20.53 |
| P10 | BC | female | left | 43 | ER+, PR+, HER2- | 16.12 |
|  | BC |  | right | 43 | ER+, PR+, HER2- |  |
| P11 | BC | female | left | 41 | unknown | 49.38 |
|  | BC |  | right | 54 | ER+, PR+, HER2- |  |
| P12 | BC | male | left | 62 | ER+, PR+, HER2- | 9.45 |
| P13 | BC | male | right | 50 | ER+, PR+, HER2- | 8.35 |
| P14 | BC | female | left | 59 | ER+, PR+, HER2- | 14.5 |
|  | BC |  | right | 61 | ER-, PR-, HER2- |  |
| P15 | BC | female | right | 61 | ER-, PR-, HER2- | 58.72 |
| P16 | BC | female | right | 66 | ER+, PR+, HER2+ | 10.79 |
| P17 | BC | female | right | 45 | ER+, PR+, HER2- | 13.48 |
| P18 | BC | female | left | 53 | unknown | 34.03 |
| P19 | BC | female | left | 60 | ER-, PR-, HER2- | 25.67 |
| P20 | BC | female | left | 34 | ER-, PR-, HER2- | 17.7 |
| P21 | BC | female | left | 49 | ER+, PR+, HER2- | 14.5 |
| P22 | BC | female | right | 50 | ER+, PR+, HER2- | 19.97 |
| P23 | OC | female | unknown | 55 | unknown | 15.85 |
|  | BC |  | right | 73 | ER+, PR+, HER2- |  |
| P24 | BC | female | right | 39 | ER+, PR+, HER2- | 35.1 |

Patient no.: patient number used in this manuscript, BC: breast cancer, OC: ovarian cancer, ER: Estrogen receptor, PR: Progesterone receptor, HER2: HER2 receptor, CanRisk-PVCP (pathogenic variant carrier probability): The patient’s probability of carrying germline pathogenic variant in *BRCA1*, *BRCA2*, *PALB2*, *CHEK2*, *ATM*, *BARD1*, *RAD51D*, *RAD51C* or *BRIP1*, estimated using the CanRisk-tool.

**Table S2:** **Variant-level and gene-level enrichment analysis**

| **variant** | **cases carrier** | **cases wildtype** | **case frequency** | **control carrier** | **control wildtype** | **control frequency** | **expected** | **enrichment** | **Beta binomial *p*_adjust_** | **significance** | **Firth regression *p*_adjust_** |
| --- | --- | --- | --- | --- | --- | --- | --- | --- | --- | --- | --- |
| *FANCM*: c.5791C>T | 1 | 119 | 8.33E-03 | 598 | 305172 | 1.96E-03 | 2.35E-01 | 4.26 | 2.26E-01 |  | NA |
| *FANCD2*: c.2715+1G>A | 1 | 119 | 8.33E-03 | 286 | 300084 | 9.52E-04 | 1.14E-01 | 8.75 | 1.38E-01 |  | . |
| *FAN1*: c.922_923del | 1 | 119 | 8.33E-03 | 92 | 305685 | 3.01E-04 | 3.61E-02 | 27.70 | 5.76E-02 | . | . |
| *FANCE*: c.1111C>T | 1 | 119 | 8.33E-03 | 15 | 305797 | 4.90E-05 | 5.89E-03 | 169.90 | 1.46E-02 | * | * |
| *MSH6*:c.2150_2153del | 1 | 119 | 8.33E-03 | 7 | 305808 | 2.29E-05 | 2.75E-03 | 364.07 | 8.77E-03 | ** | NA |
| *MSH4*: c.2198C>A | 1 | 119 | 8.33E-03 | 93 | 284742 | 3.27E-04 | 3.92E-02 | 25.52 | 5.76E-02 | . | . |
| *WRN*: c.724+1G>A | 1 | 119 | 8.33E-03 | 0 | 302863 | 0.00E+00 | 0.00E+00 | NA | 2.77E-03 | ** | * |
| *WRN*: c.2103_2104del | 1 | 119 | 8.33E-03 | 2 | 305789 | 6.54E-06 | 7.85E-04 | 1274.13 | 4.12E-03 | ** |  |
| *BLM*: c.1642C>T | 2 | 118 | 1.67E-02 | 104 | 305120 | 3.41E-04 | 4.09E-02 | 48.91 | 3.87E-03 | ** | * |
| *ERCC4*: c.1765C>T | 1 | 119 | 8.33E-03 | 106 | 305701 | 3.47E-04 | 4.16E-02 | 24.04 | 5.76E-02 | . | . |
| *MRE11*: c.1516G>T | 1 | 119 | 8.33E-03 | 29 | 305658 | 9.49E-05 | 1.14E-02 | 87.84 | 2.34E-02 | * | . |
| *XRCC4*: c.25del | 1 | 119 | 8.33E-03 | 321 | 305339 | 1.05E-03 | 1.26E-01 | 7.94 | 1.39E-01 |  | . |
| *SPIDR*: c.814C>T | 1 | 119 | 8.33E-03 | 0 | 304077 | 0.00E+00 | 0.00E+00 | NA | 2.77E-03 | ** | . |
| *PNKP*: c.1029+2T>C | 1 | 119 | 8.33E-03 | 1097 | 239560 | 4.56E-03 | 5.47E-01 | 1.83 | 4.22E-01 |  | NA |

**Table S2:** Variant-level analyses were performed using a beta binomial test and gene-level analyses using Firth regression. Note that monoallelic LPV/PVs in *MSH6* are associated with Lynch syndrome. Variants that were not included in the gene-level burden test due to higher carrier frequencies (*FANCM, PNKP*) or autosomal dominant inheritance (*MSH6*) are indicated by NA. Significance levels: ., *p*_adjust_ < 0.1; *, *p*_adjust_ < 0.05; **, *p*_adjust_ < 0.01; ***, *p*_adjust_ < 0.001.


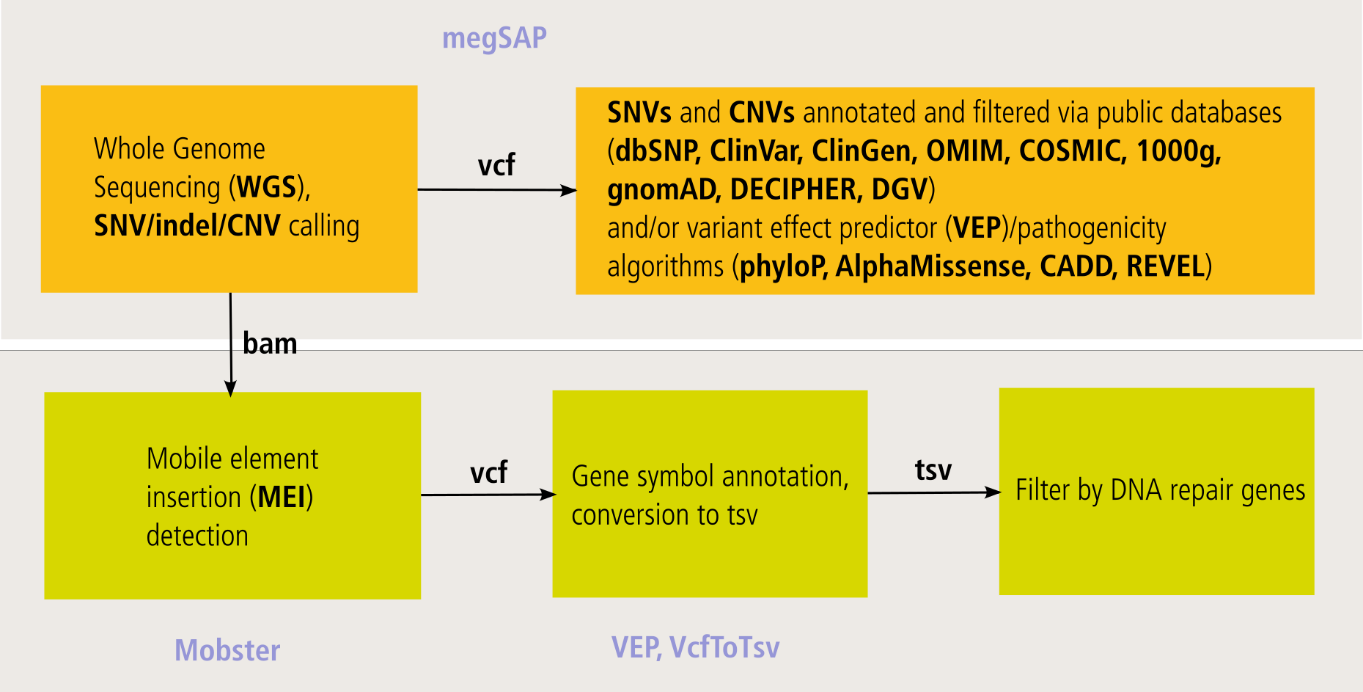


**Figure S1. Workflow of the WGS analysis.** Top panel: Variant calling and annotation using the megSAP NGS analysis pipeline, followed by variant filtering. Bottom panel: Detection of MEIs using Mobster, followed by MEI filtering based on DNA repair genes.

Databases: dbSNP^1^, ClinVar^2^, ClinGen^3^, OMIM^4^, COSMIC^5^, 1000g^6^, gnomAD^7^, VEP^8^, phyloP^9^, CADD^10^, REVEL^11^, AlphaMissense^12^, DECIPHER^13^, DGV^14^.

**
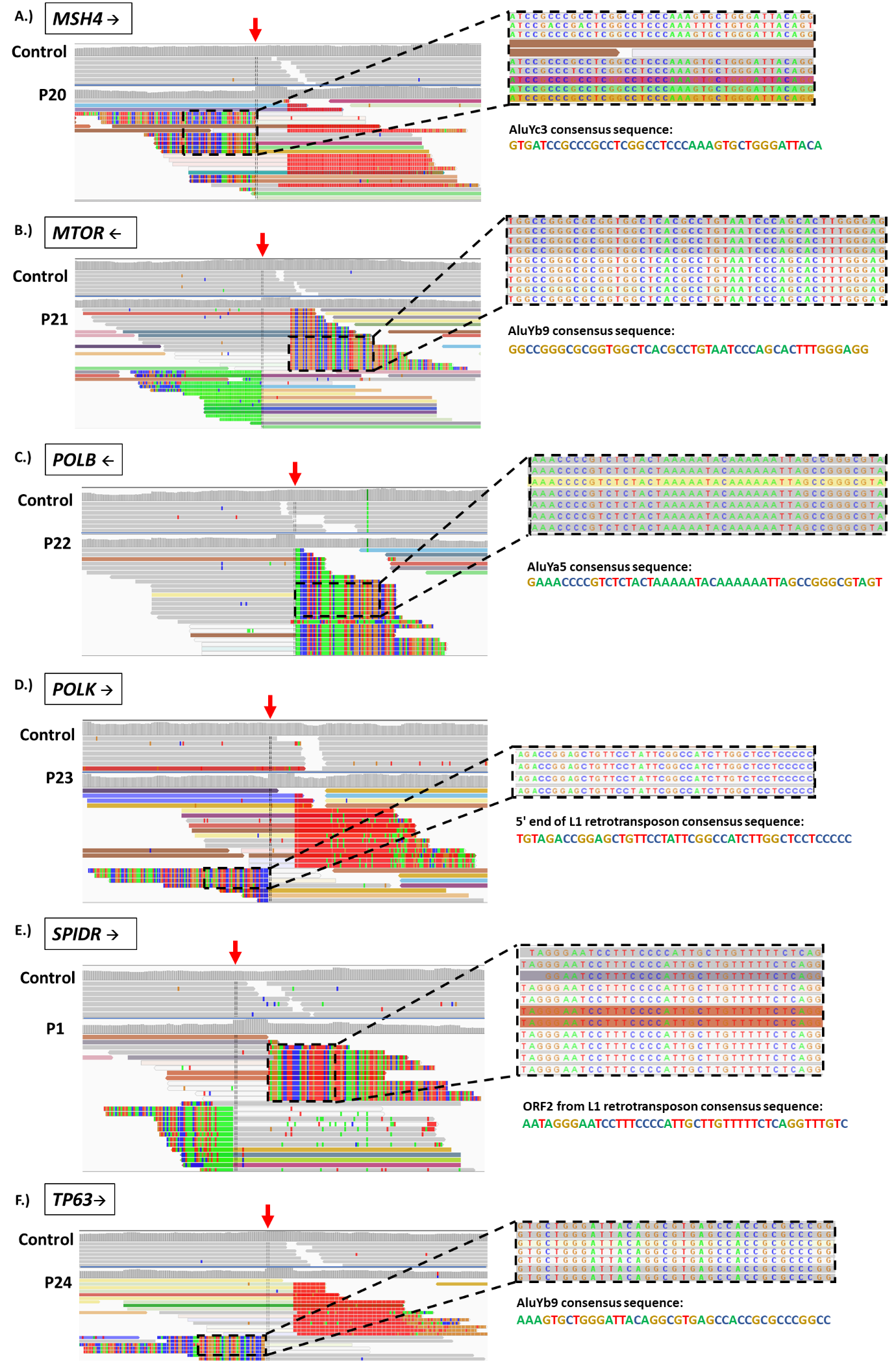
**

**Figure S2. MEIs in DNA repair genes detected by Mobster in WGS data from 134 HBOC patients.** Left side: WGS and Mobster visualization via IGV [15] comparing control (upper part) to patient (lower part) with soft-clipped based of the insertion shown. A.) AluYc3 insertion in the *MSH4* gene of patient P20. B.) AluYb9 insertion in the *MTOR* gene of patient P21. C.) AluYa5 insertion in the *POLB* gene of patient P22. D.) L1 element insertion in the *POLK* gene of patient P23. E.) L1 element insertion in the *SPIDR* gene of patient P1. F.) AluYb9 insertion in the *TP63* gene of patient P24. The red arrows mark the insertion point of each MEI.

Right side: Magnified view of the soft-clipped base sequence at the insertion point of the MEI (highlighted by a black box). Directly below each magnification is the consensus sequence of the corresponding MEI, as retrieved from the Dfam 3.7 database (<https://www.dfam.org/>).

References

1. Sherry ST, Ward MH, Kholodov M, Baker J, Phan L, Smigielski EM, Sirotkin K. dbSNP: the NCBI database of genetic variation. Nucleic Acids Res. 2001 Jan 1;29(1):308-11. doi: 10.1093/nar/29.1.308. PMID: 11125122; PMCID: PMC29783.

2. https://www.ncbi.nlm.nih.gov/clinvar; Landrum MJ, Chitipiralla S, Kaur K, Brown G, Chen C, Hart J, Hoffman D, Jang W, Liu C, Maddipatla Z, Maiti R, Mitchell J, Rezaie T, Riley G, Song G, Yang J, Ziyabari L, Russette A, Kattman BL. ClinVar: updates to support classifications of both germline and somatic variants. Nucleic Acids Res. 2024 Nov 23:gkae1090. doi: 10.1093/nar/gkae1090. [PMID:39578691]

3. https://www.clinicalgenome.org

4. Online Mendelian Inheritance in Man, OMIM®. McKusick-Nathans Institute of Genetic Medicine, Johns Hopkins University (Baltimore, MD), 2025. World Wide Web URL: https://omim.org/

5. Forbes SA, Tang G, Bindal N, Bamford S, Dawson E, Cole C, Kok CY, Jia M, Ewing R, Menzies A, Teague JW, Stratton MR, Futreal PA. COSMIC (the Catalogue of Somatic Mutations in Cancer): a resource to investigate acquired mutations in human cancer. Nucleic Acids Res. 2010 Jan;38(Database issue):D652-7. doi: 10.1093/nar/gkp995. Epub 2009 Nov 11. PMID: 19906727; PMCID: PMC2808858.

6. 1000 Genomes Project Consortium; Auton A, Brooks LD, Durbin RM, Garrison EP, Kang HM, Korbel JO, Marchini JL, McCarthy S, McVean GA, Abecasis GR. A global reference for human genetic variation. Nature. 2015 Oct 1;526(7571):68-74. doi: 10.1038/nature15393. PMID: 26432245; PMCID: PMC4750478.

7. Chen, S., Francioli, L.C., Goodrich, J.K. et al. A genomic mutational constraint map using variation in 76,156 human genomes. Nature 625, 92–100 (2024). https://doi.org/10.1038/s41586-023-06045-0

8. McLaren W, Gil L, Hunt SE, Riat HS, Ritchie GR, Thormann A, Flicek P, Cunningham F. The Ensembl Variant Effect Predictor. Genome Biology Jun 6;17(1):122. (2016) doi:10.1186/s13059-016-0974-4

9. Pollard KS, Hubisz MJ, Rosenbloom KR, Siepel A. Detection of nonneutral substitution rates on mammalian phylogenies. Genome Res. 2010 Jan;20(1):110-21. doi: 10.1101/gr.097857.109. Epub 2009 Oct 26. PMID: 19858363; PMCID: PMC2798823.

10. Philipp Rentzsch, Daniela Witten, Gregory M Cooper, Jay Shendure, Martin Kircher, CADD: predicting the deleteriousness of variants throughout the human genome, Nucleic Acids Research, Volume 47, Issue D1, 08 January 2019, Pages D886–D894, https://doi.org/10.1093/nar/gky1016

11. Ioannidis NM, Rothstein JH, Pejaver V, Middha S, McDonnell SK, Baheti S, Musolf A, Li Q, Holzinger E, Karyadi D, Cannon-Albright LA, Teerlink CC, Stanford JL, Isaacs WB, Xu J, Cooney KA, Lange EM, Schleutker J, Carpten JD, Powell IJ, Cussenot O, Cancel-Tassin G, Giles GG, MacInnis RJ, Maier C, Hsieh CL, Wiklund F, Catalona WJ, Foulkes WD, Mandal D, Eeles RA, Kote-Jarai Z, Bustamante CD, Schaid DJ, Hastie T, Ostrander EA, Bailey-Wilson JE, Radivojac P, Thibodeau SN, Whittemore AS, Sieh W. REVEL: An Ensemble Method for Predicting the Pathogenicity of Rare Missense Variants. Am J Hum Genet. 2016 Oct 6;99(4):877-885. doi: 10.1016/j.ajhg.2016.08.016. Epub 2016 Sep 22. PMID: 27666373; PMCID: PMC5065685.

12. Cheng J, Novati G, Pan J, Bycroft C, Žemgulytė A, Applebaum T, Pritzel A, Wong LH, Zielinski M, Sargeant T, Schneider RG, Senior AW, Jumper J, Hassabis D, Kohli P, Avsec Ž. Accurate proteome-wide missense variant effect prediction with AlphaMissense. Science. 2023 Sep 22;381(6664):eadg7492. doi: 10.1126/science.adg7492. Epub 2023 Sep 22. PMID: 37733863.

13. Foreman J, Perrett D, Mazaika E, Hunt SE, Ware JS, Firth HV. DECIPHER: Improving Genetic Diagnosis Through Dynamic Integration of Genomic and Clinical Data. Annu Rev Genomics Hum Genet. 2023 Aug 25;24:151-176. doi: 10.1146/annurev-genom-102822-100509. Epub 2023 Jun 7. PMID: 37285546; PMCID: PMC7615097.

14. MacDonald JR, Ziman R, Yuen RK, Feuk L, Scherer SW. The Database of Genomic Variants: a curated collection of structural variation in the human genome. Nucleic Acids Res. 2014 Jan;42(Database issue):D986-92. doi: 10.1093/nar/gkt958. Epub 2013 Oct 29. PMID: 24174537; PMCID: PMC3965079.

15. Robinson JT, Thorvaldsdóttir H, Winckler W, Guttman M, Lander ES, Getz G, Mesirov JP. Integrative genomics viewer. Nat Biotechnol. 2011 Jan;29(1):24-6. doi: 10.1038/nbt.1754. PMID: 21221095; PMCID: PMC3346182
